# Industrial Odor Source Identification Based on Wind Direction and Social Participation

**DOI:** 10.3390/ijerph16071242

**Published:** 2019-04-08

**Authors:** Mohamed Eltarkawe, Shelly Miller

**Affiliations:** Mechanical Engineering Department, University of Colorado Boulder, Sustainability, Energy and Environment Complex, East Campus, 4001 Discovery Drive, Boulder, CO 80303, USA; eltarkaw@colorado.edu

**Keywords:** odor source identification, social participation, sensory methods, wind direction, smartphone

## Abstract

Industrial odors have been a major concern in many communities in Colorado (USA). Odor source identification is important for any mitigation strategy. The aim of this work was to identify odor sources using wind direction and odor data collected by social participation. For more than one year residents reported time, date, location and description of the odor occurrence by means of a smartphone technology. The odor spatial distribution and wind roses generated from local stations were used to identify odor sources. The majority of odor reports happened in North Denver (57%) and Greeley (33%). North Denver analysis showed that a single facility that manufactures pet food was responsible for the pet food odor (the most reported odor, 81 reports). Dead animal and sewage odors were associated with a North Denver meat and grease recycling facility, and the Metro Wastewater treatment plant, respectively. Roofing tar odor was probably associated with a facility that treats crossties and utility poles with creosote. Another odor that was often described as a refinery odor was less likely to be associated with the Denver oil refinery and more likely to be associated with one of the four facilities in the northwest of Globeville that uses asphalt and creosote materials. In Greeley, most reports (133 reports) happened in LaSalle, a small town in the southern part of Greeley. All reports from LaSalle described one offensive odor that was produced by a biogas facility east of LaSalle. The feasibility of odor source identification using wind direction and social participation was demonstrated. A regional cooperation to reduce odor problems in North Denver is highly recommended.

## 1. Introduction

Under-resourced communities often experience higher concentrations of air pollution and face greater risks of health problems [1,2,3,4]. Odors from industrial sources is one type of air pollution that affects residents of these often-low-income communities physically and psychologically [5,6,7,8,9,10]. North Denver (CO, USA), which is impacted by emitted odors from the surrounding industrial facilities, is an example. Among North Denver neighborhoods, Globeville and Elyria-Swansea seem to be the most affected communities. Residents of the two neighborhoods have a long history of odor complaints. Between 2004 and 2017, Denver has received 1322 odor complaints [11,12].

The odor exposure from industrial sources within communities could lead to physical health effects and depreciation of property. More importantly, the literature has strong evidence that industrial odors are psychologically dangerous [5,9,10,13]. There is an increasing interest from local authorities as well as state level authorities in odor regulations. In response to odor and health concerns in the North Denver area, the City and County of Denver updated its odor ordinance in 2016. The ordinance requires some industries (e.g., pet food factories and marijuana production facilities) to develop Odor Control Plans (OCPs). The ordinance also extends the period during which complaints must be received in order for the City and County of Denver to trigger enforcement. The ordinance states that a facility that receives five complaints from individuals representing separate households during a period of 30 days will be required to develop an OCP [14]. Clearly to enforce the odor ordinance and assess whether the OCPs are effective, odor source identification is essential.

In response to residents’ complaints, a study was conducted specifically on asphalt odors in Globeville in 2015 [15]. A follow up study in 2017 investigated the impact of industrial odors on the residents of North Denver and four other communities. The study found an association between industrial odor exposure and wellbeing levels. The study also found that North Denver and Greeley were the most impacted communities by industrial odors among the five communities [13]. Unlike Denver, odor regulations have not been enforced in Greeley.

Odor source identification by linking spatial odor data to wind direction data at the time of odor occurrence has been used in previous studies [15,16]. Types of odor are often classified based on the odor source into two categories: odors from animal feeding operations and odors from municipal, commercial and industrial sources. Regardless of the odor type classification, there are many important factors that determine the odor impact (odor annoyance). Some of these factors are meteorological conditions, distance between the source and the receptor, and the nature of the odor which can be described by odor intensity, frequency, and hedonic tone [17].

Odors are evaluated by either instrumental or sensory methods. The instrumental methods measure the chemical compounds in the air by using for example a gas chromatography (GC) or photoionization detectors (PID). The sensory methods are a common way to assess odors by using a human panel. The main disadvantage of the instrumental methods is that some odors are perceived by the human nose below the detection limit of these instruments. In addition, accurate instrumental methods such as GC are quite expensive and cumbersome to deploy in situ. Therefore, the sensory methods have been often favorable due to their low cost, and no-detection-limit issue [18,19,20,21,22,23,24,25,26,27].

Sensory methods are widely accepted, and they provide reliable and valid information to evaluate environmental odors [27]. Locations of odor assessment by conventional sensory methods are usually pre-determined during the study design. This could limit the ability of understanding of odor spatial distribution in the study area. The impact of this limitation is often minimized by carefully selecting the odor assessment locations. Another limitation is that odor data from sensory methods are collected during a daily time-window that is small compared to the 24-hour window of daily deployment as detailed in the procedure of the Oregon Department of Environmental Quality (DEQ) [28] study and the follow up study by Eckmann et al. [16] (see Supplementary Material File A1).

Sensory methods can be conducted by dynamic olfactometry as well as by using social participation [29]. Dynamic olfactometry is used to quantitatively evaluate odor concentrations by providing accurate and controlled dilutions of odor samples from the source site with odorless air to reduce the odor concentration to its detection threshold. The main disadvantage of sensory methods of odor field inspection is the cost, specifically when a large number of panelists are recruited to evaluate odors for a long period of time [29]. Social participation has proven to be reliable in addition to its advantage of positive psychological impact on the affected community by involving residents in the odor assessment studies [29,30,31]. Social participation can be used to identify odor sources [30].

The use of smartphone technology is rapidly increasing. It was estimated that over 2.1 billion smartphone users in 2016 and this number is expected to reach 2.87 billion by 2020 [32]. In addition to many other research fields, this rapid advancement in the smartphone technology has opened new opportunities for researchers to collect more data from public reporting. Using smartphone technology to collect odor data will overcome the location and time limitations, so the panelists will be able to report odors at any time and from any location. This technology will provide less expensive, and valuable spatial data about odors in the study area. In addition, positive psychological impact from the social participation will be a benefit to the community [31].

The objective of this study is to identify industrial odor sources in North Denver, and Greeley Colorado using wind data and odor reports collected by social participation. The motivation for this study was because anecdotal reports of health and wellbeing impacts have been reported for many years and our most recent study showed a significant association between odor exposure and subjective well-being. For the same five communities, the study found that residents who reported that the air was fresh in their area, they also experienced higher levels of general satisfaction and happiness [13]. Our hypothesis is that an odor source can be identified by using the location of an odor report downwind and the wind direction at the time of the report.

## 2. Study Area, Materials, and Methods

This section describes the methodology that was used to identify odor sources in the study area. The study area and potential odor sources are described (Section 2.1), followed by information about odor perception and recording (Section 2.2). Wind data (Section 2.3) and odor sensitivity test (Section 2.4) are presented. The last Section 2.5 presents data preparation and analysis.

### 2.1. Odor Sources, and Study Area

The area of Globeville and Elyria Swansea in North Denver contains over 70% commercial and industrial businesses. Odor issues have been a major concern for the residents. Some of those businesses are Purina (a pet food factory), Suncor Energy (a major refinery), Koppers Inc. (a creosote wood treatment facility), Altogether Recycling, METech Recycling, Owens Corning Denver Roofing Plant, Owens Corning Trumbull Asphalt Plant, Metro Wastewater Reclamation District, Cobitco Inc. (an asphalt emulsion company), National Western Stock Show Association, and DARPRO Solutions (a meat/grease/cooking oil recycling facility). Some of these businesses, such as the Metro Wastewater Reclamation facility, are located as far as 1.6 km from the closest residential area, while other businesses are just across the street from residential homes, such as Purina. The point source emission inventory 2014 provided emission information for some of these businesses such as emission rates of some of the pollutants and number of emission units on each site. The point source inventory was obtained from the Air Pollution Control Division of the Colorado Department of Public Health and Environment (CDPHE) as well as DDPHE. In addition to commercial businesses, Globeville and Elyria-Swansea are divided by railroad tracks and two major highways (I-70 and I-25 which carry about 150,000 and 250,000 vehicles daily, respectively). 

The North Denver area in this study includes all neighborhoods from Denver County and Adams County that contain or are close to these types of commercial and industrial businesses. Four other locations from Colorado were selected for comparison with North Denver. Three of the selected locations are demographically similar to Globeville and Elyria Swansea where the population is predominantly Hispanic, with low household income and education. The three locations are Greeley, Fort Lupton and Pueblo. A fourth location which is Fort Collins was also selected to be demographically different than the other locations. Table 1 show the demographic information for the five locations. Greeley, Fort Collins and Fort Lupton are located in the north of Colorado and surrounded by farms and agriculture lands. The three locations are known for manure odor from livestock farms existing in the area due to the presence of many meat packing plants including one of the world largest meat production companies, JBS in in the northeast side of the Greeley. Pueblo is the largest city in the south of Colorado with no known history of odor issues.

### 2.2. Odor Perception and Recording

A reporting tool was designed to collect data from the five Colorado communities. The reporting tool was an android smartphone application (SPA) named *Report Odor* [33]. Alternatively, a web-based link was implemented for non-Android smartphone users. The reporting tool allowed participants to report odors within their communities at any time and from any location. It collected odor description, time, date, and location of the odor occurrence. The collected information was saved on a secure server. A copy of every report from the SPA was simultaneously sent to the Denver Department of Public Health and Environment (DDPHE), which is the entity that oversees odor complaints in the City and County of Denver. The co-operation with DDPHE was as a result of efforts from the nonprofit organization Groundwork Denver so that the odor reports could lead to action in reducing odors in North Denver. Using the SPA, participants from North Denver could choose to make the report an official complaint by providing additional information (name, phone number and address). The other four communities (Greeley, Fort Collins, Fort Lupton, and Pueblo) are located outside of the City and County of Denver where odor ordinances are not enforced at this moment. The reporting tool provided a list of odor descriptions that was developed based on the odor complaints that the DDPHE received during recent years. In case the participant experienced an odor that was not on the list, the reporting tool allowed the participant to type in a description of the odor or simply select other. The list included: pet food, natural gas, vehicle exhaust, roofing tar, mothballs/creosote, sewage, dead animals/rendering, livestock, refinery, rotten eggs, marijuana, paint/paint thinner and other.

Recruiting for this study was based on the methods from our companion study described elsewhere [13] in which participants (n = 326) were asked to submit an online subjective well-being and odors survey and to provide their email addresses at the end of the survey. Every participant who completed this online survey and provided an email address was contacted and asked to use the smartphone application or the alternative web-based reporting link. A $5 gift card was sent to the physical addresses of those who successfully completed the online survey and submitted a test odor report. Voluntarily, the participants were asked to respond to periodically sent reminders asking them to report air condition in their area. In this case, participants could select whether the air was odorous or fresh.

### 2.3. Wind Data

Odor reports were collected from the five communities between 10 March 2016 and 19 April 2017. Since the number of odor reports was small in Fort Collins, Fort Lupton and Pueblo, wind analysis were considered only in North Denver and Greeley. The time of odor occurrence submitted in each report was used to collect wind data (wind speed and wind direction) from the closest air monitoring station. Hourly wind speed and direction are available on the CDPHE’s website.

In North Denver study area, wind speed and direction were collected from two air monitoring stations: the CAMP station (Air Quality System number, AQS#: 080310002) which is located in downtown Denver at the intersection of 21st Street and Broadway and the I-25 Globeville (I25 Glob) station (AQS#: 080310028, 4905 N. Acoma Street) which is located in the heart of Globeville near the I-25 Highway. In Greeley study area, the wind speed and direction were collected from the Weld County Tower station, (AQS#: 081230009, 3101 35th Avenue, Greeley, CO 80634) which is located within Greeley City’s limits.

The wind data from I-25 Globeville station, which is more representative of the study area, became available online starting from 1 November 2016. Therefore, for the period from 10 March 2016 to end of October 2016, wind data were collected from CAMP (73% of the reports).

The hourly wind speed and direction were also collected for the entire study period so a general picture of wind conditions can be established in the study area. In North Denver, CAMP and I-25 Globeville stations were used while the Weld County Tower was used for wind data in Greeley. In Denver, winds seemed to be mostly low (94% of wind speed measurements were less than 4 m/sec). About 16% of wind measurements were calm defined as 0.5 m/sec or less. The prevailing wind direction in North Denver was from SSW, consistent with the wind analysis of Morgan et al. [15]. Slightly higher winds were experienced in Greeley: 85% of the measurements were less than 4 m/sec (light breeze to gentle breeze based on the Beaufort scale) and only 1.7% of the measurements were clam (see Figure S1 in the Supplementary Material).

### 2.4. Odor Sensitivity Test

To achieve reliability and more reproducible results, it is recommended that the panelists participating in an odor study have an acceptable level of odor sensitivity. An odor sensitivity test described in the American Society of the International Association for Testing and Materials (ASTM) methods (E679-91) was performed on a randomly selected group of 25 participants [34]. The objective of the sensitivity odor test which uses the psychophysical methodology of the ascending 3-Alternative Forced-Choice (3-AFC) method, is to measure odor detection thresholds for the selected group. Our team which consisted of a test operator and an assistant, used the St. Croix Sensory’s odor sensitivity test kit (St. Croix Sensory, Inc., Stillwater, MN, USA). The 30-minute tests were conducted at public libraries in North Denver and Greeley. The results showed that the odor detection thresholds for all selected participants were within the acceptable range recommended by St. Croix.

### 2.5. Data Preparation and Analysis

Every odor report was submitted with a location (a physical address or intersection) that was converted into northing and easting coordinates. The spatial odor data was visualized via the Geographic Information System (GIS). To protect the privacy of the participants, Transportation Analysis Zones (TAZs) were used to generate color gradient maps instead of point-address analysis. TAZs are geography units widely used in transportation planning models. Like census blocks, the size of TAZs is quite small in urban areas but it is much bigger in rural areas. For each odor report, wind speed and direction were collected using the time and date of submission. Wind data were used to generate wind roses. The combination of the spatial odor distribution and wind roses was used to identify odor sources in North Denver and Greeley where most of the reports were registered. Our hypothesis is that the wind direction at the time of reporting and the location of the odor report downwind can be used to identify the odor source. In other words, if the odor reports from a suspected source happened to appear on one side (e.g., east to the source), it is anticipated that the frequency of wind direction during these reports will be mainly from the other side (e.g., west direction). For a systematic method for estimating wind direction frequency, an imaginary line was drawn between the possible source and the location of TAZs with high reporting. The wind direction frequency was calculated within a quadrant (45-degree clock-wise and 45-degree counter-clockwise from the imaginary line).

## 3. Results

This section is organized as follows: First, an overview of the odor reports in the five communities is presented (Section 3.1), then the results of North Denver odors and source identification is presented (Section 3.2), followed by the results and odor source identification in Greeley (Section 3.3).

### 3.1. An Overview of Odor Reports in the Five Communities

During the period from March 2016 to April 2017, a total number of 476 reports were received from the five communities. Most of those reports were from North Denver and Greeley. LaSalle is a small town in the southern part of Greeley. The number of reports from LaSalle alone was 133 reports, 28% of the total number of reports from the five communities. Other communities sent much less odor reports so they were excluded from subsequent analysis. Table 2 shows that pet food, refinery, roofing tar, dead animals/rendering, and sewage were frequently reported odors in North Denver while in Greeley, residents often reported putrid, sewage, chemical and livestock odors.

### 3.2. Odors in North Denver

After an overview of the most reported odors and their potential sources in North Denver (Section 3.2.1), pet food odor (Section 3.2.2), roofing tar odor (Section 3.2.3), and refinery odor (Section 3.2.4) are described with potential sources. Then dead animal odor (Section 3.2.5), and sewage odor (Section 3.2.6) and their potential sources are presented.

#### 3.2.1. An Overview of Odor Reports in North Denver

**Figure 1:**
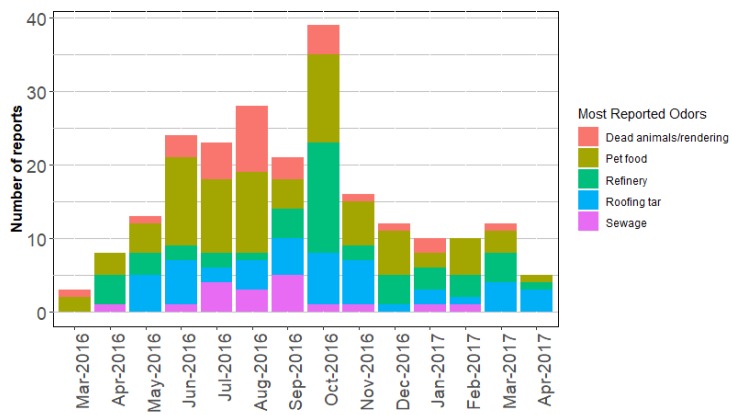
Monthly odor report from March 2016 to April 2017 in North Denver. Only the most-report-odors in North Denver are included.

Figure 1 shows the monthly reporting from March 2016 to April 2017 for the most reported odors in North Denver. The monthly reporting was higher between May 2016 and October 2016, then dropped significantly after October 2016.

In the following analysis for North Denver area, the *most-reported-odors* refer to: pet food, refinery, roofing tar, dead animals/rendering, and sewage. Table 3 shows the possible source for each of these odors in North Denver, the total volatile organic compounds (TVOCs) emissions, the number of emission units, and the estimated weekly work hours for the emission units in each facility based on the point source emission inventory of industries [15]. The table also shows the distance in kilometer between the facility and the two air monitoring stations that are located in North Denver.

**Figure 2:**
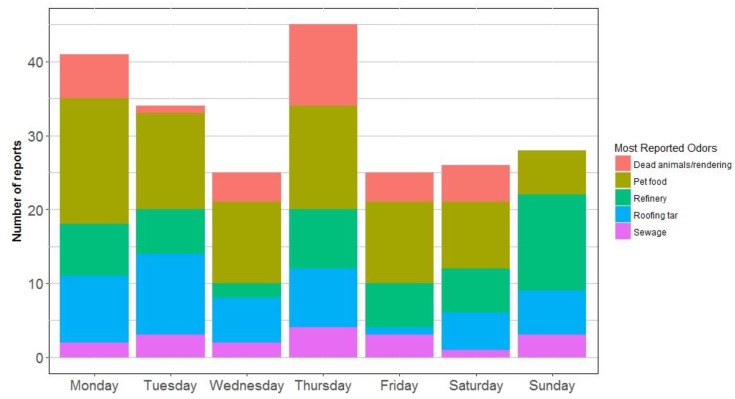
The most-report-odors in North Denver, by day of the week.

Figure 2 shows day-of-the-week odor reporting for the most-reported-odors in North Denver. The graph shows that the most-reported-odors happened every day except for dead animal odors. The absence of dead animal odor during Sundays is in partial agreement with the percentage of weekly work hours of emission units at Darling International Inc., which is the possible source for dead animal odor as shown in Table 3. Darling International Inc. has nine emission units that work about 33% of the time every week.

#### 3.2.2. Pet Food Odor

**Figure 3:**
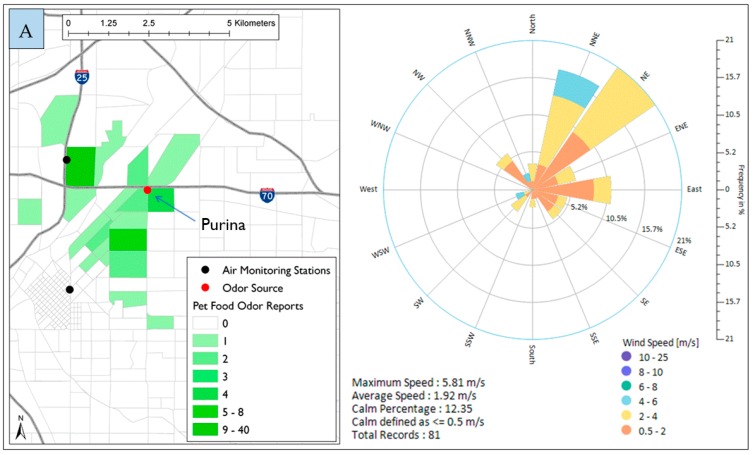

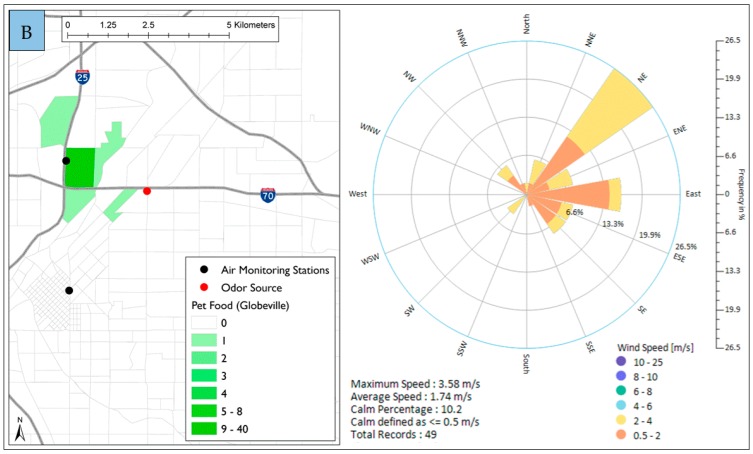

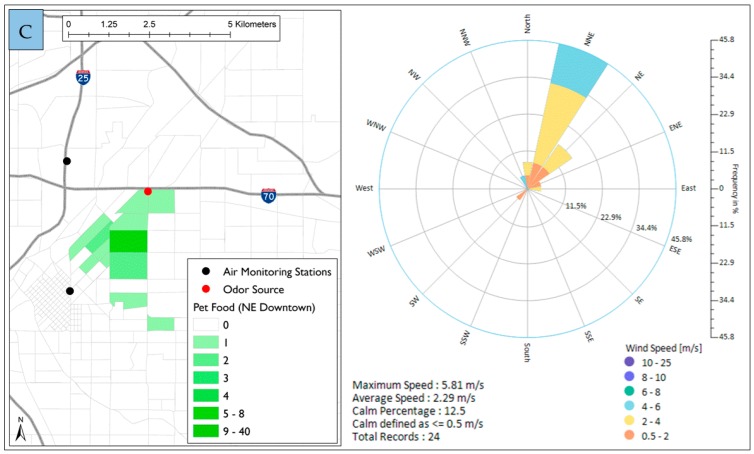
Spatial distribution of pet food odor reports in North Denver and wind roses during the time of reporting. (**A**) all odor reports; (**B**) reports from Globeville area; (**C**) reports from Northeast downtown.

The most reported odor in North Denver was pet food odor likely associated with Nestle Purina Pet Care Company. The combination of wind direction and odor data was used to identify the odor source. Figure 3A shows the location of Nestle Purina Company marked in red and all pet food odor reports (81 reports) represented per the color gradient map. Pet food reports were mainly from Globeville, Elyria-Swansea, and northeast of Downtown Denver, and came from 39 unique addresses indicating that some of these unique addresses reported odors more than one time. 

There were 49 pet food reports from Globeville area (Figure 3B) which is to the west of Nestle Purina. As it was anticipated, 76% of the frequency of the wind direction during the 49 reports was coming from the east direction, more specifically, between NE and SE. Similarly, when considering reports from northeast Downtown Denver, 88% of the wind direction frequency blew between NNW and ENE direction which is the direction of the source location as shown in Figure 3C.

#### 3.2.3. Roofing Tar Odor

**Figure 4:**
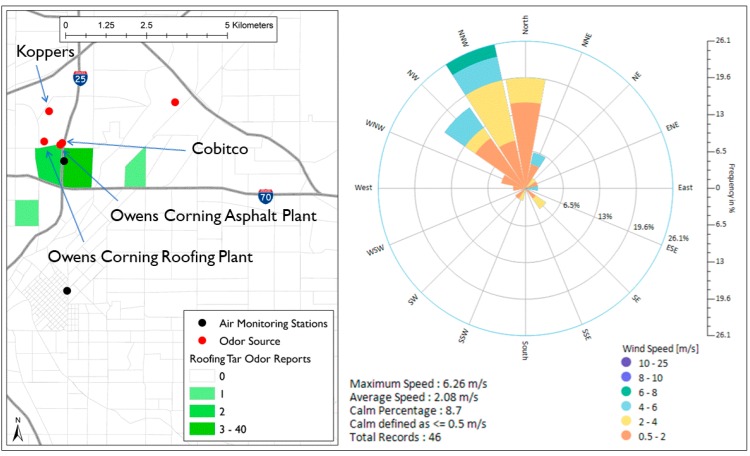
Spatial distribution of roofing tar odor reports in North Denver and wind roses during the time of reporting.

Roofing tar odor was often reported in North Denver. A total of 46 reports were received during the study time. There are four possible sources of roofing tar odor in North Denver: Owens Corning Denver Roofing Plant, Owens Corning Trumbull Asphalt Plant, Koppers Industries Inc., and Cobitco Inc. [15]. Figure 4 shows the location of the four facilities as well as the roofing tar odor reporting locations. During the time of all the 46 reports, 80% of the wind direction frequency was coming from WNW–NNE direction consistent with the location of the four facilities.

#### 3.2.4. Refinery Odor

**Figure 5:**
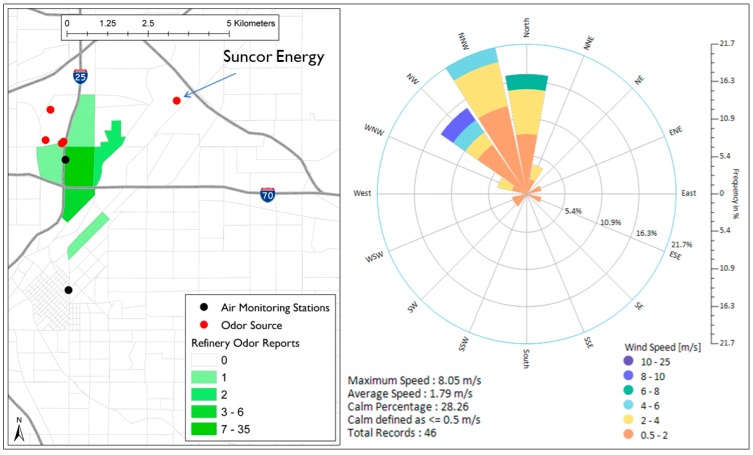
Spatial distribution of refinery odor reports in North Denver and wind roses during the time of reporting.

Another frequently reported odor was the refinery odor. The possible source of this odor is Suncor Energy, a major refinery to the east of Elyria-Swansea. When wind direction frequency during the refinery odor reports was calculated, 37% of the wind direction frequency blew from the northeast quadrant (NE), which is the direction of Suncor Energy. However, 81% of the wind direction frequency was coming from WNW–NNE direction suggesting that the refinery odor that was reported in North Denver might not be associated with Suncor Energy only. As we can see in Figure 5, 46 (98% of total) refinery odor reports were documented in Globeville and North Denver downtown. During the time of these reports, the wind direction was mostly from the northwest direction (81% of the wind frequency was coming from WNW–NNE direction). This indicates that one or more of the four facilities located in northeast area is a possible source of the refinery odor.

#### 3.2.5. Dead Animal Odor

**Figure 6:**
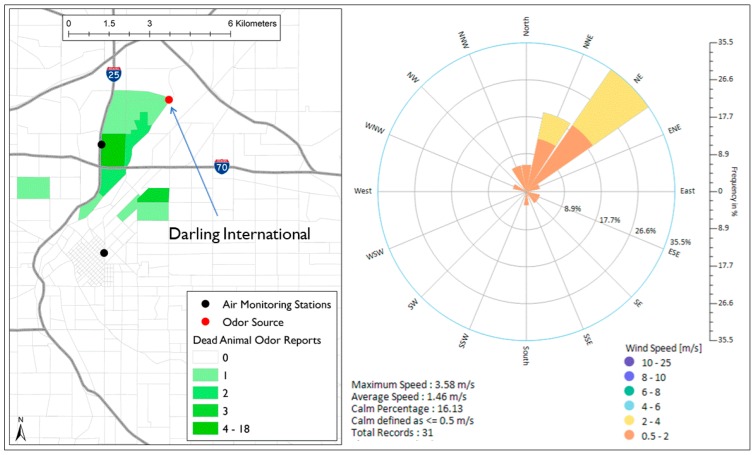
Spatial distribution of dead animal odor reports in North Denver and wind roses during the time of reporting.

Dead animal odor is another strong odor in North Denver that was reported 31 times during the study time. The possible source of this odor is Darling International Inc., which is located to the north of Globeville and Elyria-Swansea (Figure 6). The wind roses show that 77% of the wind direction frequency during all the 31 reports blew between NNW and ENE direction suggesting that Darling International Inc. is a possible source of most of the dead animal odor in North Denver.

#### 3.2.6. Sewage Odor

**Figure 7:**
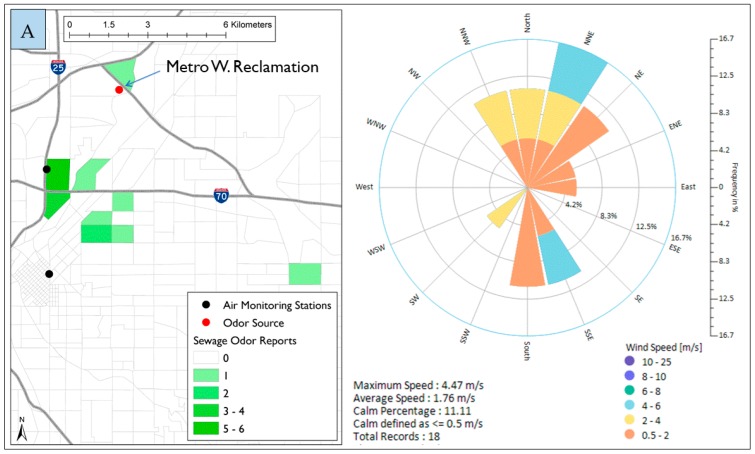

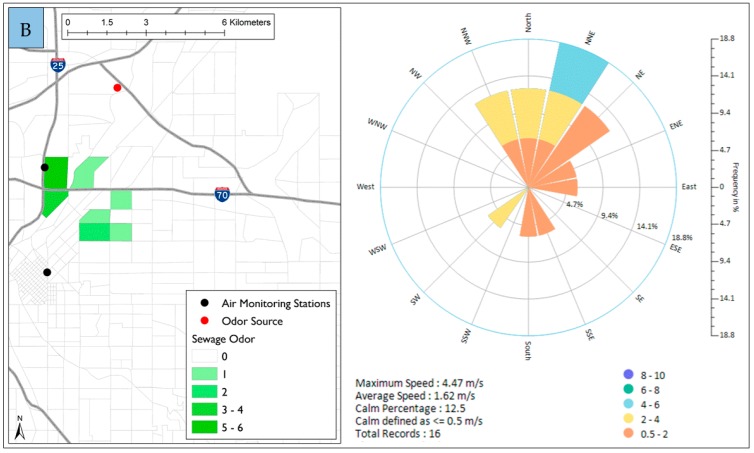
Spatial distribution of sewage odor reports in North Denver and wind roses during the time of reporting. (**A**) all odor reports; (**B**) reports from Globeville area and northeast downtown

The sewage odor was reported only 18 times during the study. A possible source is the Metro Wastewater Reclamation District located to the north of Elyria-Swansea just outside Denver City and County boundaries. Figure 7A shows the location of Metro Wastewater Reclamation and it also shows that most of the reports happened in Globeville and northeast downtown. 

When considering only reports from Globeville and northeast downtown (Figure 7B), the frequency of the wind direction was mostly from the north (59% came from NW–NE direction). This suggests that the Metro Wastewater Reclamation District is a possible source of the odor. It is worth noting that during the 16 reports of sewage odor reported in Globeville, about 20% of the wind direction frequency was blowing from the south direction, precisely between SE and SW. This probably indicates that more efforts are required to identify other possible sources.

### 3.3. Odors in Greeley

**Figure 8:**
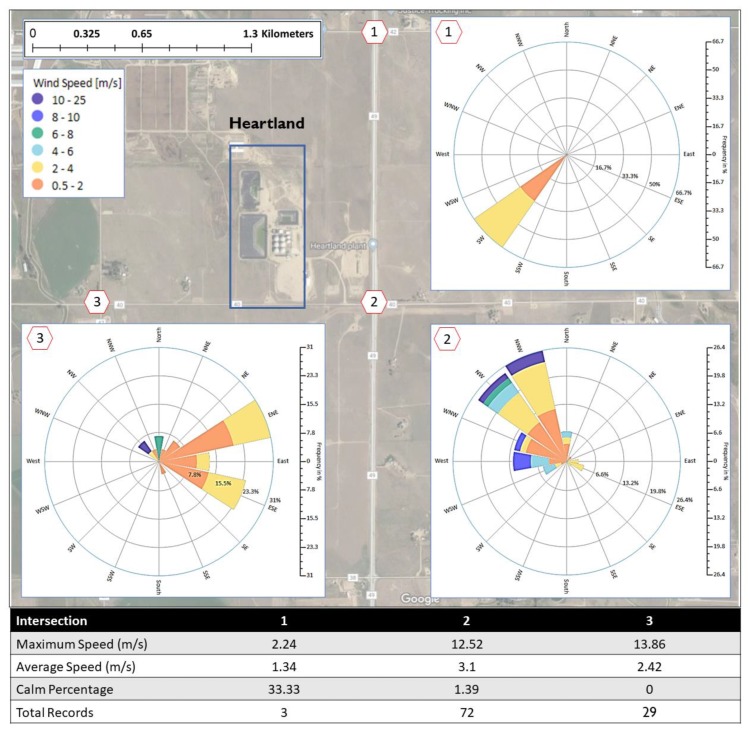
Location of Heartland Biogas facility, the possible source of putrid/sewage/chemical odor in LaSalle and the wind roses at the intersections near the facility during the time of odor reporting.

The odor reports received from Greeley represent 33% (157 reports) of total number of reports in the study. Most (85%, 133 reports) were received from LaSalle. All reports from LaSalle described the odor as a putrid, chemical, or sewage odor. The possible source of these three odors is Heartland Biogas, a $115 million biogas facility east of LaSalle town. The distance between the biogas facility and the Weld County Tower is 15.5 kilometer. The facility is capable of converting approximately 1000 tons of food waste and cattle manure into renewable natural gas every day [35]. The facility is surrounded by a few ranch homes where residents seemed to be impacted by the odor emitted during the process of converting cattle manure and food waste into natural gas. It is worth mentioning that the 133 reports received form LaSalle were submitted by 10 people. During the study, residents from this area mostly reported odors at road intersections near the Heartland facility, precisely at the northeast, southeast, or southwest intersection labeled as 1, 2, and 3 respectively in Figure 8. 

A very limited number of reports were associated with residents’ physical addresses. To protect the privacy of the residents, the reports from physical addresses were assigned to the closest intersection from the three intersections 1, 2, or 3. Figure 8 also shows the location of Heartland Biogas and the wind roses at each intersection during the reported odors. Only three reports occurred at intersection 1. The wind direction frequency shows that the wind blew from the southwest direction during these three reports. At the intersection 2, wind direction frequency was mainly from northwest during the time of 72 reports that were documented at this location. At intersection 3, there were 30 reports during which wind blew mostly from east-northeast. These results show that Heartland Biogas is the most probable source responsible for the odor that was often described as a putrid, chemical or sewage odor. It is important to mention that a total of 28 reports of putrid, chemical and sewage were excluded from the above analyses because they were submitted without clear addresses.

## 4. Discussion

The feasibility of odor source identification using wind direction and social participation was demonstrated. The odor data from social participation were collected using smartphone technology. The wind roses were generated from monitoring stations data during the time of reporting to identify the odor sources in North Denver and Greeley where most of the odor reports were registered. This method was used in number of previous studies [15,16,30].

### 4.1. North Denver Odor Data Analysis

The results of our study showed that pet food, sewage, and dead animal odors were associated with Nestle Purina Pet Care Company, Metro Wastewater Reclamation District, and Darling International Inc. respectively. During the time of odor reporting related to these three facilities, there was a strong association between wind directions and source locations. Each of these odors is distinctive therefore most residents were able to easily identify the source of odor.

At least one of the four facilities located to the northwest of Globeville (Owens Corning Denver Roofing Plant, Owens Corning Trumbull Asphalt Plant, Koppers Industries Inc., and Cobitco Inc.) was responsible for much of the roofing tar odors. This is in a good agreement with Morgan et al. [15], who found that all tar/asphalt odor events occurred during NNW winds and they considered the same four facilities for investigation as likely sources for tar/asphalt odor. Their GC-MC analysis suggested that Koppers Inc. was responsible for much of the tar/asphalt odor. [15].

Our results, however, did show that residents reported another odor—referred to as refinery odor—which from our analysis appeared less likely to be associated with Suncor Energy and more likely to be associated with one of the four facilities in the NW. When we look closely at all residents who reported roofing tar and/or refinery in our study, we found that about 50% of residents reported both odors. The other 50% reported only roofing tar or only refinery odor. The group of residents that reported both odors was responsible for 91% of roofing tar odor reports and 85% of refinery odor reports. In other words, most of odor reports related to roofing tar and refinery were documented by a group of residents that experienced the two different odors. The reporting tool provides a list of odor descriptions including roofing tar and refinery. It is probably the residents tended to select refinery odor from the reporting tool because the odor was distinctive from roofing tar odor. However, more investigations are required to identify which facility was responsible for the refinery odor.

The impact of odor source on a nearby community depends on many factors. One factor is the nature of the odor source. This includes the odor hedonic tone (pleasant or unpleasant), the intensity of the odor, and when the odor is released. Offensive odors that are emitted in a large amount are more likely to have a greater impact. Odors released during the day in summer time could have more impact than those are emitted during a windy night when most people are indoor. Figure 1 shows high odor reports between May and October 2016. This is most likely associated with the time spent per day outdoors during summer and early fall seasons. A second factor is meteorological conditions. As shown in our analysis, a residential community that are located downwind of an odor source is more likely to be impacted by odors. The fact that the prevailing winds in North Denver were from SSW probably maximize the impact of the Purina factory as it is located to the south of Elyria-Swansea residential area while possibly minimized the impact of other facilities since they are located north to the residential area. Another very important factor is the distance between the source and the impacted location. Odor impact is often maximized near the source. Our analysis showed that one sewage odor report was registered at 9.09 km from its possible source as shown in Figure 7A. This cannot be ascertained more precisely. The rest of reports for all odor types happened at less than 6.00 km from their possible sources. In fact, all other sewage reports registered within 4.06 km from M.W. Reclamation. The distance between Purina and the furthest pet food report was 4.01km. The distance between the furthest roofing tar report and the four potential sources ranged from 2.38 to 3.30 km. The furthest dead animal report was registered at 5.65 km.

One of the challenges facing the City and County of Denver is that all these odor-emitting facilities (except Purina) that affect North Denver residents are actually located in Adams County just outside City and County of Denver’s limits. In May 2016, DDPHE, the entity that oversees odor complaints in the City and County of Denver, updated the odor ordinance which is expected to reduce odor complaints from North Denver residential area. The ordinance requires facilities that exceed five odor complaints in 30 days, exceed dilution threshold standards, or fall within certain industry types to develop odor control plans [14]. The fact that most of odor sources are located outside City and County of Denver where Denver’s regulations cannot be enforced highlights the importance of more regional cooperation regarding the odor issue in North Denver.

### 4.2. Greeley Odor Data Analysis

Most of odor reports in Greeley occurred near the Heartland biogas facility. Results showed that this facility was responsible for the odor reported by the nearby residents. Wind roses generated during the time of reporting clearly showed that Heartland was the source of the putrid, sewage and chemical odors. Short after the facility started operating in 2015, residents were complaining about the emitted odors during the process of energy production. The facility was demanded by Weld county commissioners to cease operations in December 2016 due to the odor complaints as well as other issues. The facility sued Weld County over the shutdown and continued to operate until Spring 2017 when it was shut down and still not operating until the time of publication. The odor reports recorded in our study were consistent with the operation time of the Heartland facility. Considering the exact location of reporting, all reports of putrid, chemical, or sewage were registered within 2.14 kilometers from Heartland biogas. As we can see from the hourly winds during the entire study period (Supplementary Materials Figure S1), the northwest quadrant was responsible for 29% of wind frequency consistent with the high number of reports happening at the southeast intersection. In this study we did not validate the odor type. We did observe that there most like was some confusion between two odor types: refinery and roofing tar. In future studies this set may be warranted. We did do a sensitivity test of a subset of the study participants to understand whether they were more sensitive to odor, but we did not find that was the case.

## 5. Conclusions

The aim of this work was to identify odor sources in five communities in Colorado; Greeley, Fort Collins, Fort Lupton, North Denver, and Pueblo. From 10 March 2016 to 19 April 2017, residents from the five communities sent 476 odor reports using smartphone technology and an alternative web-based online link. For the same period, wind direction and wind speed were collected from air monitoring station in or near the reporting area. Most of odor reports were received from North Denver (57%) and Greeley (33%). Other communities reported much less odor reports; Fort Lupton (5%), Pueblo (4%) and Fort Collins (1%).

In North Denver, Pet food odor was the most reported odor. Some other odors were frequently reported in North Denver such as roofing tar, refinery, dead animal and sewage. The odor spatial analysis combined with wind roses generated during the time of reporting in North Denver showed that pet food odor was associated with Nestle Purina Pet Care, the dead animal odor was associated with Darling International Inc., and the sewage odor was associated with the Metro Wastewater Reclamation District. The location of these facilities and uniqueness of their odors helped residents in providing accurate data. Wind roses generated during the time of reporting showed that winds mostly blew from the direction of these sources.

Other odors in North Denver were less distinctive, chemical-like, and emitted by facilities that are located relatively close to each other. Four facilities located to the northwest of Globeville (Owens Corning Denver Roofing Plant, Owens Corning Trumbull Asphalt Plant, Koppers Industries Inc., and Cobitco Inc.) seemed to be responsible for the roofing tar odor and the refinery odor. Previous work showed that roofing tar odor (tar/asphalt odor) was associated with Koppers Inc. Our work has shown that beside the roofing tar odor, another odor was frequently reported in North Denver. Even though this odor was described as a refinery odor, our analysis showed that it was less likely to be associated with Suncor Energy and more likely to be associated with one of the four facilities in the northwest of Globeville. More detailed investigation is probably required to determine the source of the refinery odor among the four possible sources.

In Greeley, a large number of odor reports were received about putrid, sewage, chemical or livestock. The number of reports from LaSalle (a small town in the southern part of Greeley) alone was 133 reports, 28% of the total number of reports from the five communities. Odor report data and wind rose analysis in Greeley showed that the odor that was often described as putrid, sewage or chemical in LaSalle was produced by Heartland Biogas Facility located east of LaSalle.

Many studies have found that industrial facilities and factories similar to those investigated in our study produce high emissions of VOCs and odorous compounds [36,37] supported by other studies that found high concentrations of VOCs near industrial locations [38]. We recommend conducting VOC measurements near these facilities, specially during the upcoming redevelopment projects for better mitigation plans.

The data provided in our study are crucial in solving the odor issues that many residents have in the state of Colorado and our methods could be used in other locations having similar experiences. Because bad odors are associated with a lower sense of wellbeing, a focus on resolving and better understanding odor impacts is warranted.

## Figures and Tables

**Table 1:** Demographic information of the five communities.

Communities	Population	Median Household Income (k)	Average Household Size	Hispanic (%)	Bachelor or Higher (%)
Denver *	600,158	$50.3 k	2.22	31.8	42.9
Globeville	3360	$26.5 k		68.7	11.2
Elyria Swansea	6940	$33.8 k		81.8	11.3
Greeley City	92,889	$46.3 k	2.63	36.0	25.8
Fort Lupton City	7377	$50.2 k	3.09	55.0	8.9
Pueblo City	106,595	$34.7 k	2.37	49.8	19.7
Fort Collins City	143,986	$53.8 k	2.37	10.1	51.9

* Denver data presented here are for the entire county of Denver, but the study focuses on the northern neighborhoods of Denver.

**Table 2:** Number of odor reports for each odor type in five communities in Colorado; Denver, Fort Collins, Fort Lupton, Greeley and Pueblo.

Odor Types	Denver	Fort Collins	Fort Lupton	Greeley	Pueblo
Dead Animals	31	0	0	1	0
Livestock	4	1	3	9	0
Marijuana	9	0	0	0	1
Mothballs	2	0	0	0	0
Natural gas	3	0	1	0	0
Pet food	81	0	0	0	0
Refinery	48	0	0	0	0
Roofing tar	46	0	0	0	0
Rotten eggs	5	0	0	0	0
Sewage	18	0	1	61	0
Vehicle exhaust	8	0	2	5	3
Chemical	0	0	0	48	0
Putrid	0	0	0	15	0
Other	7	1	2	12	8
No odor	7	2	17	6	8
Total	269	4	26	157	20

**Table 3:** Denver’s most-reported-odors, their possible sources, the estimated VOC emissions, number of emission units and the percentage of weekly work hours of the emission units.

Reported Odor	Possible Odor Source	Distance to I-25 Glob Station (km)	Distance to CAMP Station (km)	VOC Estimated Emission (tpy)	Total of Emission Units	% of Weekly Work Hours
**Pet food**	Nestle Purina Pet Care Co.	2.5	3.9	1.2	78	46%
**Refinery**	Suncor Energy—Denver Refinery	4.0	6.7	421.6	275	98%
**Roofing tar**	Cobitco Inc.—an asphalt emulsion Co.	0.8	4.6	N/A	N/A	N/A
**Roofing tar**	Koppers Industries Inc.	1.7	5.6	3.3	9	78%
**Roofing tar**	Owens Corning Trumbull Asphalt Plt.	0.7	4.5	25.9	29	97%
**Roofing tar**	Owens Corning Denver Roofing Plant	0.8	4.5	21.8	23	96%
**DeadAnl.**	Darling International Inc.	2.8	5.9	3.7	9	33%
**Sewage**	Metro Wastewater Reclamation Dist.	3.7	6.8	16.3	20	85%

## Supplementary Materials

The following are available online at https://www.mdpi.com/1660-4601/16/7/1242/s1. Supplementary File A1: The total time estimated for odor measurement in the Oregon Department of Environmental Quality study. Figure S1: Wind roses generated from hourly wind data in North Denver and Greeley from 10 March 2016 to 19 April 2017.
