# Supplementary material for: Industrial Odor Source Identification Based on Wind Direction and Social Participation
Source: Int J Environ Res Public Health. 2019 Apr 8;16(7):1242. doi: 10.3390/ijerph16071242 (PMC6479448; doi:10.3390/ijerph16071242)
Supplement: Supplementary file 1 [file ijerph-16-01242-s001.pdf]

Supplementary File A1: Total Time Estimated for Odor Measurement in the Oregon Department of Environmental Quality Study:

The DEQ study conducted a total of 760 visits during one year during which there were 24 positive detection visits. Intuitively, this means there were 736 negative detection visits (no odor was detected).

$$negative\ visits = 760\ total - 24\ positive\ visits = 736\ negative\ visits$$

When odor was not present, the team (staff) spent 5 minutes at the site. Therefore,

$$time\ spent\ during\ negative\ visits = 736 * 5 = 3680\ minutes$$

We know from the DEQ study that the team spent total time of 187 minutes when odor was present during the 24 positive visits. An average of 8 minutes duration for each detected odor episode. Therefore,

$$Total\ time\ spent\ (unsuccessful\ and\ positive) = 3680\ +\ 187\ =\ 3867\ \text{minutes}$$

This amount of time is equivalent to less than 3 days.
Total time visits in days = 3867/60 = 64.5 hours = 2.7 days
The represents small fraction of total time of the one-year study time.
Percentage of visits time = 2.7 days/ 365 days = 0.7%
The DEQ study stated that the total time panelist spent to evaluate odors was 10213 minutes (an average of 13.4 minute per visit which was rather equivalent to the maximum time was recorded).

Figure A1: Wind roses generated from hourly wind data in North Denver and Greeley from March 10th, 2016 to April 19th, 2017:

Wind roses generated from hourly wind data from March 10th, 2016 to April 19th, 2017. Left: Wind data from the CAMP and the I-25 Globeville stations in North Denver. Right: Wind data from the Weld County Tower station in Greeley. Top (Denver), bottom (Greeley).

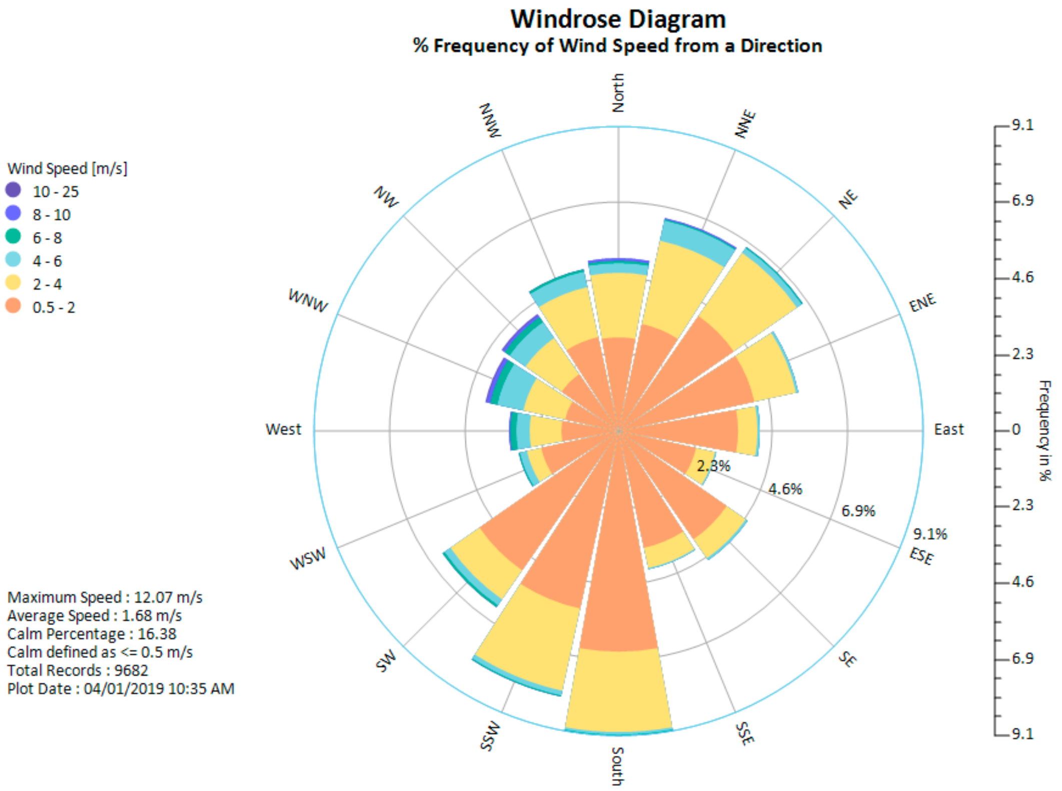
Windrose Diagram
% Frequency of Wind Speed from a Direction
Wind Speed [m/s]
10 - 25
8 - 10
6 - 8
4 - 6
2 - 4
0.5 - 2
North
NNE
NE
ENE
East
ESE
SE
SSE
South
SSW
SW
WSW
West
WNW
NW
NNW
2.3%
4.6%
6.9%
9.1%
Frequency in %
9.1
6.9
4.6
2.3
0
2.3
4.6
6.9
9.1
Maximum Speed : 12.07 m/s
Average Speed : 1.68 m/s
Calm Percentage : 16.38
Calm defined as <= 0.5 m/s
Total Records : 9682
Plot Date : 04/01/2019 10:35 AM

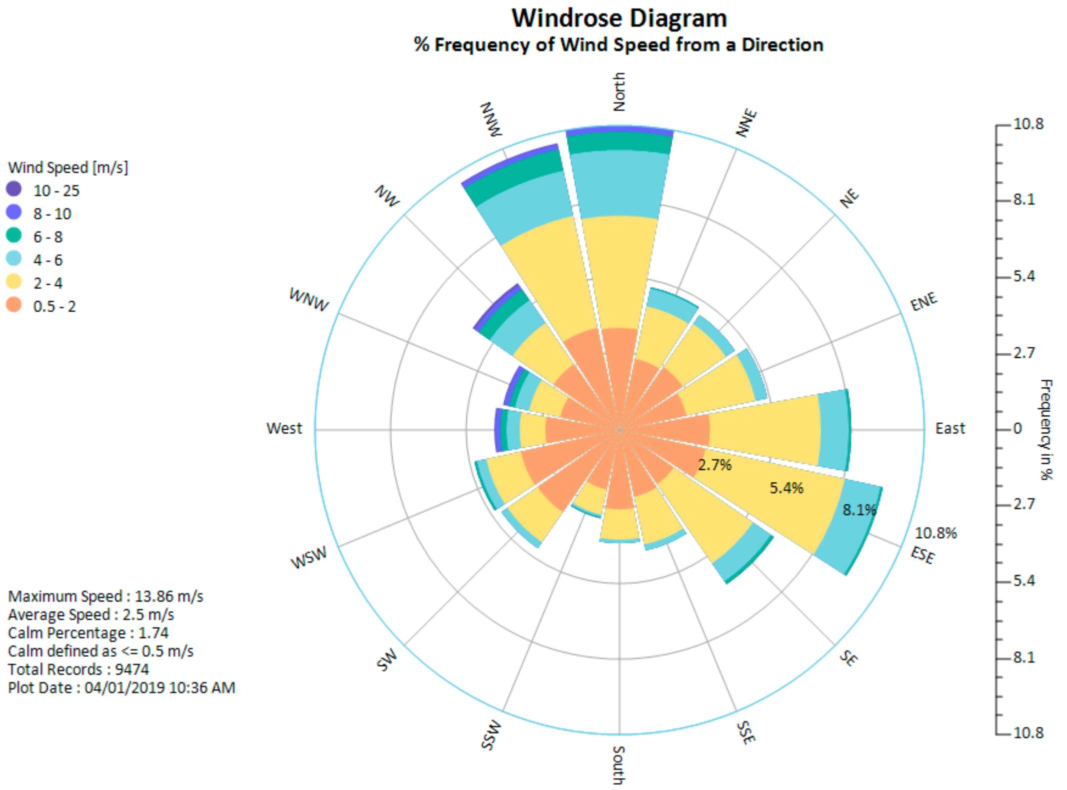
Windrose Diagram
% Frequency of Wind Speed from a Direction
Wind Speed [m/s]
10 - 25
8 - 10
6 - 8
4 - 6
2 - 4
0.5 - 2
North
NNE
NE
ENE
East
ESE
SE
SSE
South
SSW
SW
WSW
West
WNW
NW
NNW
2.7%
5.4%
8.1%
10.8%
Frequency in %
10.8
8.1
5.4
2.7
0
2.7
5.4
8.1
10.8
Maximum Speed : 13.86 m/s
Average Speed : 2.5 m/s
Calm Percentage : 1.74
Calm defined as <= 0.5 m/s
Total Records : 9474
Plot Date : 04/01/2019 10:36 AM
